# Approaches in Physical Activity: From Basic to Applied Research

**DOI:** 10.1155/2016/6498624

**Published:** 2016-12-04

**Authors:** Julien S. Baker, Leonardo dos Santos, Bruce Davies, Emmanuel G. Ciolac, Danilo S. Bocalini

**Affiliations:** ^1^Institute for Clinical Exercise & Health Science, University of the West of Scotland, Paisley, UK; ^2^Department of Physiological Sciences, Federal University of Espirito Santo, Vitoria, ES, Brazil; ^3^Health and Exercise Science Research Unit, University of South Wales, Treforest, Pontypridd, UK; ^4^Exercise and Chronic Disease Research Laboratory, Physical Education Department, School of Sciences, São Paulo State University, Bauru, SP, Brazil; ^5^Translational Physiology Laboratory, Department of Physical Education and Aging Sciences, São Judas Tadeu University, São Paulo, SP, Brazil

Changes in modern lifestyles, including diets high in salt, sugar, and undesirable fats and decreases in physical activity levels, have contributed to the increasing incidence and prevalence of chronic diseases. Several nonpharmacological strategies have been developed aimed at promoting a healthy lifestyle. These strategies have aimed to reduce medication, increase the quality of life, and enhance longevity. Restructuring human lifestyle is a process that should be encouraged in all ages, by all genders, and in all sectors related to health care provision. Strategies focusing on lifestyle intervention and the impacts that physical activity and exercise have on populations have been widely researched over previous decades.

As a result of these studies, many recommendations outlining the benefits of exercise and physical activity have been published. Unfortunately, optimal exercise prescription and physical activity recommendations for different clinical pathologies and conditions related to cardiovascular disease are not well established. This is particularly true if we consider the unique and individual scenario of each pathology and patient.

Measures of physical fitness, most notably cardiorespiratory fitness, are considered to be an important marker of health status or risk for cardiovascular disease (CVD) and early mortality. Therefore, the functional status of individuals and all related components should be monitored in human populations globally.

Although physical fitness is in part genetically determined, it can be improved by regular and appropriate physical activity. In most physical activity recommendations, increasing physical activity levels of sedentary individuals is often the focus. The objective is to provide the greatest improvement in health status while providing protection against CVD risk and early mortality. Evidence suggests that cardiorespiratory fitness rather than physical activity may provide the most robust protection against CVD risk and early mortality [1–8].

Studies have shown that higher levels of cardiorespiratory fitness are inversely associated with a reduced cardiovascular disease (CVD) risk profile in both children and adolescents [1, 2, 5, 9, 10]. Regarding the other two health-related physical fitness components, that is, muscular fitness and speed/agility [11], there is a lack of available studies which have examined the independent associations of these components with CVD risk factors [4], although evidence is accumulating [12, 13].

Ortega and colleagues examined the associations between objectively measured physical activity and cardiorespiratory fitness and a number of CVD risk factors including blood pressure and five metabolic parameters (fasting levels of insulin, glucose, triglycerides, total cholesterol, and HDL-C). There were nearly 1150 participants involved of near-equal gender, aged between 9-10 yrs and 15-16 yrs from Sweden and Estonia. The authors demonstrated that cardiorespiratory fitness measures were more strongly related to CVD risk factors than physical activity at both ages while suggesting that associations become stronger as participants reach adolescence. Measures of cardiorespiratory fitness exhibited stronger relationship (*p* < 0.05) with waist circumference (WC) compared to physical activity objectively measured (*p* > 0.05) in children and adolescents independently of sex, age, and height [1]. Participants showing a high cardiorespiratory fitness also have a lower prevalence of being overweight or obese, excess of total fat, or having a high-risk waist circumference independent of age and gender and physical activity levels, suggesting a role for genetics in cardiorespiratory fitness.

Investigations have also shown that young people with high cardiorespiratory fitness levels have a lower body mass index, WC, % of body fat [2] and lower resting levels of C-reactive protein [7, 10]. Anderssen et al. [9] examined the cardiorespiratory fitness levels of nearly 3000 European youth aged either 9 or 15 years and found that those within the lowest quintile of fitness, in comparison to those in the highest quintile, were 13 times more likely to exhibit high levels of traditional CVD risk factors (total cholesterol/high-density lipoprotein cholesterol ratio, plasma triglycerides, insulin resistance, sum of four skinfolds, and systolic blood pressure), independent of age group and gender. These investigations support the need for a high cardiorespiratory fitness level as a means of reducing CVD risk in youth. Some have suggested that engagement in vigorous physical activity may be necessary in order to ensure that elements of physical fitness are enhanced in youth [1, 4, 11, 14].

Additionally, muscular fitness can be defined as the capacity to work against a given resistance. However, the capacity of individuals to generate maximum force depends on numerous factors such training status, gender, and the nature of activity performed and thereby cannot be measured by one single test [11]. Jump tests are common methods used in youth populations to provide a measure of muscular power. Although few studies have examined the association between muscular power and CVD risk in youth, some evidence shows a negative association between jumping performance and CVD risk.

In a study by Artero and colleagues, the independent association between muscular or cardiorespiratory fitness and clustered metabolic risk was examined in a cohort of 709 children and adolescents of mixed gender [13]. From their findings the authors demonstrated that muscular fitness was negatively associated with clustered metabolic risk independently of cardiorespiratory fitness (*β* = −0.249, *p* < 0.001). Again, individuals within the lowest fitness quartile exhibited a higher clustered metabolic risk than those in the highest quartile, reinforcing the need of a high physical fitness as a means of reducing CVD risk.

Similar findings have already been reported four years earlier [15] when muscular fitness was defined as an index computed from the standardized scores of explosive strength, endurance strength, and maximal handgrip strength. In that study, the authors examined whether the amount of physical activity and physical fitness (i.e., aerobic capacity and muscle strength) in European adolescents could influence metabolic risk. Although no relationship was noted between physical activity and metabolic risk, favorable relationships between metabolic risk and muscular strength or cardiorespiratory fitness were seen in both male and female groups. Specifically, greater cardiorespiratory fitness in males and greater muscle strength in females were associated with a healthy metabolic risk profile. In 2009, this concept was reinforced by a study demonstrating that muscular strength was negatively associated with clustered metabolic risk, independent of cardiorespiratory fitness [12].

In addition, some evidence is available, although limited, suggesting that improving speed and or agility may be important for improving cardiovascular health [4, 16]. As a fitness parameter, speed has been defined as the ability to move the body as fast as possible whereas agility has been defined as the ability to move quickly and change direction while maintaining balance and control, thereby comprising speed, balance, power, and coordination [11]. From their recent review, Ruiz et al. [4] were able to identify only one qualitative study examining the association between speed/agility and CVD risk factors: in the study by Twisk et al. [16] the authors produced an index of neuromotor fitness from measures of muscular strength, flexibility, speed of movement, and coordination and found an inverse association between measures of adiposity (sum of four skinfolds). Nonetheless, no association was evident for the other metabolic CVD risk factors measured (total serum cholesterol, HDL-C, and the TC: HDL ratio). Currently, the association between speed/agility and CVD risk has been largely ignored in youth populations and, as a result, its use as an indicator of present health status or a predictor of future health status is unclear.

It is undeniable that, for children and young people, the school environment affords an ideal setting to practice health-promoting behaviors and is widely recognized as an important setting for intervention [8, 17, 18]. Moreover, this setting provides a vehicle whereby interventions can reach a large number of individuals from assorted socioeconomic surroundings, avoiding stigmatization while affording an environment for youth to partake in physical activity. Nonetheless, it is important that practitioners devise activities that are appropriate to improve the health and well-being of youth. This is especially significant as physical education may be the only opportunity for some to engage in any form of vigorous physical activity.

Actually, a growing body of evidence has demonstrated that adaptations typically associated with traditional endurance exercise interventions may also occur using low volume, high-intensity interval training (HIIT) [19–21]. As lack of time is commonly cited as a key determinant of being physically inactive regardless of age, gender, or ethnicity, the role of HIIT has been purported as a plausible alternative to traditional endurance based activities for improving the health and well-being of individuals.

Because original HIIT protocols have been designed for use in a laboratory [19–21], one of the challenges faced was to devise a protocol that could be undertaken by a large number of individuals at the same time, which was not labour or time intensive, expensive, or difficult to implement. Thus, the school based HIIT protocol, discussed elsewhere [22–24], was established to determine its effects upon measures of health and well-being, including physical fitness parameters.

Notwithstanding, there is a need for development of effective exercise programs and behavioral lifestyle interventions to reduce damage, improve function, or treat problems associated with particular pathological conditions. Based on the translational perspective, evidence was sought showing morphological, biochemical, and physiological benefits, as well as the molecular aspects in both experimental models from basic to clinical and applied sciences.

This special issue was proposed to combine biology, biomechanics, and behavior sciences at the human level. The findings presented here can be used to improve rehabilitation and prescription of physical activity, not only for health promotion, but also for sports performance, and are also important for the progression of science in this field of knowledge. The editors considered the interdisciplinary nature of the papers as necessary to enlarge the concepts from basic research to applied science.

Finally, we feel that this is a unique opportunity to provide different views in relation to biological and behavioral studies, presented in the same publication. We also feel that this publication should be available, not only to the researchers and professionals examining physical activity and its outcomes, but also in the disciplines of nutrition, medicine, pediatrics, physiotherapy, physical education, and public health.

*Julien S. Baker* *Leonardo dos Santos* *Bruce Davies* *Emmanuel G. Ciolac* *Danilo S. Bocalini*
